# Adolescent Support for Tobacco Control Policies and Associations with Tobacco Denormalization Beliefs and Harm Perceptions

**DOI:** 10.3390/ijerph16010147

**Published:** 2019-01-07

**Authors:** Jianjiu Chen, Sai Yin Ho, Lok Tung Leung, Man Ping Wang, Tai Hing Lam

**Affiliations:** 1 School of Public Health, University of Hong Kong, Hong Kong, China; chenjianjiu@gmail.com (J.C.); lloktung@connect.hku.hk (L.T.L.); hrmrlth@hku.hk (T.H.L.); 2 School of Nursing, University of Hong Kong, Hong Kong, China; mpwang@hku.hk

**Keywords:** tobacco control, policy, public opinion, tobacco denormalization

## Abstract

Public support is crucial for advancing tobacco control policies. We investigated adolescent support for such policies, and its association with potential factors of social denormalization (SD) beliefs of smoking, tobacco industry denormalization (TID) beliefs (negative perceptions of the industry), and harm perceptions of smoking. In a cross-sectional survey in Hong Kong, 13,964 secondary school students (mean age 15.0 years, 51.3% boys) reported their support (yes/no) for each of 14 tobacco control policies (e.g., further increase tobacco tax). Tobacco-related beliefs and perceptions, and smoking status were also measured. Support for the 14 tobacco control policies ranged from 17.6% to 54.1%. In current non-smokers, SD beliefs, TID beliefs, and harm perceptions were all associated with support for all tobacco control policies. In current smokers, the study factors were each associated with support for two to three policies. To conclude, support for tobacco control policies was weak to moderate in Hong Kong adolescents. SD beliefs, TID beliefs, and harm perceptions of smoking were associated with policy support in current non-smokers. In current smokers, the corresponding associations were less consistent or weaker.

## 1. Introduction

Public support is crucial for advancing tobacco control policies. Strong public support in polls and signature petitions have helped achieve legislative successes [1,2]. Conversely, some failures were partly due to inadequate public support, leaving lawmakers more susceptible to influencing by the tobacco industry [3]. Inadequate public support was also partly to blame for poor compliance to smokefree laws [4,5]. The tobacco industry (TI), recognizing the importance of public opinions, has exploited various tactics to mobilize public resistance to tobacco control policies [6,7].

An important task in tobacco control is to garner public support for policies. Social denormalization (SD) is an established approach that shifts the social norms around smoking towards being an abnormal and undesirable behaviour [8]. Tobacco industry denormalization (TID) is an underused approach that exposes the industry’s deceitful and manipulative tactics [9]. While tobacco denormalization, including SD and TID, has been found to have some favorable effects [8,9], its association with support for tobacco control policies has been studied in only two papers. In the International Tobacco Control Policy Evaluation Survey (ITC), a four-country study done in Australia, Canada, the United Kingdom (UK), and the United States (U.S.), SD beliefs and the belief that the industry should take responsibility for the harms caused by tobacco were both associated with support for industry regulation [10]. In a U.S. study, Niederdeppe et al. combined the data of a nation-wide tobacco survey with market-level anti-smoking advertisement exposure data, and found that the volume of anti-industry advertisements on the market level was associated with support for indoor smoking bans, but not tobacco advertising bans [11].

In addition, a few papers examined the association of policy support with the knowledge or the communication of the harms of tobacco. Such knowledge was associated with support for smokefree policies in two U.S. surveys [12,13], and general support for industry regulation in the ITC four-country study [10]. In another U.S. survey, self-reported exposure to media campaigns on the harms of tobacco was associated with support for smokefree policies [14]. In the study by Niederdeppe et al., the volume of advertisements on the harms of tobacco on the market-level was associated with support for indoor smoking bans, but not tobacco advertising bans [11]. 

Of the above literature, most of them studied smokefree policies or general indicators of policy support; one paper included support for advertising bans as an outcome [11]. As many countries have already implemented indoor smokefree laws [15], research on other specific policy options are increasingly being needed. Moreover, to date, only two studies have explored the association of tobacco denormalization with policy support, and with inconsistent results [10,11]. 

Another research gap is that studies on public support for tobacco control have rarely included adolescents, despite their strategic importance in determining future progress in tobacco control, as generations of adolescents become adults with voting power. If weak policy support is found in adolescents in a region, the tobacco control advocates should reflect on the reasons and take measures to garner adolescent support. 

Hong Kong, a special administrative region of China, has achieved considerable success in tobacco control [16]. Tobacco tax has increased multiple times since 1983, and now constitutes 64% of the retail price (HKD 38/59; 1 USD = 7.8 HKD) of major cigarette brands. The tax has not changed since a small increase in 2014. Since 2007, smoking in public indoor places has been banned. Six forms of graphic health warnings covering 50% of cigarette packets were introduced in 2007. Since June 2018, the number of forms of graphic warnings has increased to 12, and the size to 85%. 

The present study aimed to investigate (i) adolescent support for various tobacco control policies in Hong Kong, (ii) the associations of socio-demographic and other smoking-related characteristics with policy support, and (iii) the associations of SD beliefs, TID beliefs, and harm perceptions of smoking with policy support. The policy options in the present study covered extensions of smokefree areas, tobacco packaging regulations, tobacco promotion restrictions, tax increases, combating illicit trade, and endgame. These policies were considered locally relevant, but had not been implemented or could be strengthened at the time of data collection.

## 2. Methods

### 2.1. Data Source 

A cross-sectional survey using a random sample of secondary schools stratified by all 18 districts of Hong Kong was conducted in 2014–2015. The survey was conducted in classrooms during school hours, using anonymous pencil-and-paper questionnaires. All students in the schools were invited. A total of 41,035 questionnaires were returned from 92 schools, with student- and school-level response rates of 95% and 36% [17]. 

To reduce the burden of completing a long questionnaire, our survey questionnaire had three versions, with each school assigned one version by random. The questionnaires comprised common core items and version-specific items. The present analysis used data from the version with the required variables completed by 14,414 students in 31 schools. Ethics approval was granted by the Institutional Review Board of the University of Hong Kong/Hospital Authority Hong Kong West Cluster (UW 14-487).

### 2.2. Variables

SD beliefs were measured by two questions. The first question, “How many Hong Kong people do you think would accept smoking?”, had response options (score) of “none (4)”, “some (3)”, “half (2)”, “most (1)”, and “all (0)”. The second question, “Do you think the social acceptance of smoking in Hong Kong has changed in recent years?”, had response options (score) of “increasingly accepted (0)”, “no change/not sure (1)”, and “increasingly unaccepted (2)”. SD beliefs were analyzed as the sum of the two scores (0–6), with greater scores indicating stronger SD beliefs. 

TID beliefs were also measured by two questions: “Do you think the tobacco industry is respectable?”, and “Do you think the tobacco industry tries to get youth to smoke?”. Both questions had the same options of “definitely yes”, “probably yes”, “probably no”, and “definitely no”, which were recoded into 0, 1, 2, and 3, respectively, for the first question; and 3, 2, 1, and 0, respectively, for the second. TID beliefs were analyzed as the sum of the two scores (0–6), with greater scores indicating stronger TID beliefs.

Harm perceptions of smoking were measured by one question, “Do you think smoking will harm your health?”, with options (score) of “definitely not (0)”, “probably not (1)”, “probably will (2)”, and “definitely will (3)”. 

Students reported their support (yes/no) for each of 14 tobacco control policies: (1) Ban smoking within 5 meters from the entrance of a building; (2) ban smoking at transport waiting areas; (3) ban smoking around children in public places; (4) ban smoking at home when a child is present; (5) ban smoking in a private car when a child is present; (6) show health warning texts and pictures on at least 75% of cigarette packet surface, and ban the display of trademarks; (7) stronger health warning pictures on cigarette packets; (8) change the health warning pictures on cigarette packets every 1-2 years; (9) show smoking cessation hotlines on cigarette packets; (10) ban the display of tobacco products at point of sale; (11) ban smoking scenes in movies which people under 18 can watch; (12) ban smoking in people born since 2010; (13) further increase tobacco tax; (14) step up efforts in combating illicit cigarettes. Students also reported whether they had ever smoked (even one puff) and the number of days they smoked in the past 30 days (0 day/1–2/3–5/6–9/10–19/20–29/30). Those who did and did not smoke in the past 30 days were classified as current smokers and current non-smokers. Current non-smokers were further categorized as “never smokers” and “ever, non-current smokers”.

The other covariates were sex, age (in years), perceived family affluence (relatively poor/poor to average/average/average to rich/relatively rich), peer smoking (none/less than half/half or more), and the number of co-residing smokers (0/1/≥2). 

### 2.3. Analysis

After excluding 450 questionnaires with internal logical inconsistencies, with missing data for over half of the questionnaire items, or with missing data for any of the variables used in this study, 13,964 (96.9%) remained for analysis. 

The proportions of adolescents who supported the 14 tobacco control policies were calculated in all and subgroups stratified by sex, age (≤13 years old/14–15/≥16), and smoking status (never/ever, non-current/current). These calculations and all other descriptive analyses were weighted by age, sex, and grade, based on the population characteristics provided by the Education Bureau. 

We examined the associations of the total number of tobacco control policies supported (continuous outcome) with sex, age, perceived family affluence, peer smoking, and the number of co-residing smokers (study factors). These associations were examined in current non-smokers and current smokers separately, first without covariate adjustment, and then with adjustment of covariates that were deemed confounders. The covariates selected for adjustment, if any, are shown together with the results of the analysis. 

We also examined the associations of SD beliefs, TID beliefs, and harm perceptions of smoking (study factors) with support for each of the 14 tobacco control policies (binary outcomes) and with the total number of policies supported (continuous outcome), with mutual adjustment of the study factors. These associations were separately examined in current non-smokers and current smokers. We also adjusted for age, sex, perceived family affluence, peer smoking, the number of co-residing smokers, and smoking (“never” vs. “ever, non-current”; in current non-smokers only).

The above analyses of associations were stratified to produce results that would be of value for populations with different smoking prevalence. The interaction effects between study factors and current smoking were also tested.

For binary outcomes, Poisson regression models with robust variance estimators were used [18]; for continuous outcomes, linear regression models were used. All models were adjusted for a potential school clustering effect. *p* values less than 0.05 were considered statistically significant. All analyses used STATA 13.1 (StataCorp, College Station, TX, USA).

## 3. Results

Table 1 shows that the students had a mean age (standard deviation) of 15.0 (1.8) years, and 51.3% were boys. The prevalence rates of “current” and “ever” smoking were 5.4% and 14.3%. The mean (standard deviation) of the SD beliefs score was 3.5 (1.1), and the corresponding figures for TID beliefs and harm perceptions of smoking were 3.8 (1.3) and 2.9 (1.5). The mean (standard deviation) of the total number of tobacco control policies students supported was 4.8 (4.6).

Table 2 shows that students’ support for the five smokefree policies ranged from 34.9% (banning smoking at home when a child is present) to 54.1% (banning smoking at transport waiting areas). For the four packaging policies, the support ranged from 23.6% (changing the health warning pictures on cigarette packets every 1–2 years) to 35.0% (showing smoking cessation hotlines on cigarette packets). For the other five policies, the support ranged from 17.6% (banning smoking scenes in movies which people under 18 can watch) to 49.7% (stepping up efforts in combating illicit cigarettes). Girls showed stronger support than boys for 11 policies, while boys showed stronger support for one policy (Ps < 0.05). Support generally decreased with age for nine policies, but increased with age for one policy (Ps < 0.05). For all 14 policies, “never” smokers showed stronger support than “ever, non-current” smokers, who in turn showed stronger support than “current” smokers (Ps < 0.001). 

Table 3 shows that, in general, the total number of tobacco control policies supported was associated with female sex and having fewer co-residing smokers in both current non-smokers and current smokers. The total number of policies supported was also associated with having fewer smoking peers in current non-smokers, but not in current smokers. The interaction effects between peer smoking and current smoking were significant (Ps < 0.001). Age and perceived family affluence were generally not associated with the total number of policies supported in current non-smokers or in current smokers.

Table 4 shows that, in current non-smokers, SD beliefs, TID beliefs, and harm perceptions of smoking were all associated with support for all of the 14 tobacco control policies. In current smokers, however, the three study factors were each positively associated with support for only two to three policies (SD beliefs were inversely associated with support for one). For SD beliefs, the strongest association was observed for “banning smoking in people born since 2010” (prevalence ratio (PR) 1.14) in current non-smokers, and “banning smoking within 5 m from the entrance of a building” (PR 1.27) in current smokers. For TID beliefs, the association with support for “banning smoking scenes in movies which people under 18 can watch” was the strongest in both current non-smokers (PR 1.27) and current smokers (PR 1.22). For harm perceptions of smoking, the strongest association observed was for “banning smoking around children in public places” (PR 1.35) in current non-smokers, and “banning smoking at home when a child is present” (PR 1.43) in current smokers. Of the 42 PRs in current smokers, 35 PRs favored policy support (PR > 1), but only seven were statistically significant. Nonetheless, many of the non-significant PRs in current smokers were comparable to the PRs in current non-smokers (e.g., PRs of support for “banning smoking within 5 m from the entrance of a building” in relation to TID beliefs: 1.11, 0.91–1.36 (current smokers) vs. 1.12, 1.11–1.14 (current non-smokers)). The interaction effects between the three study factors and current smoking in relation to the 14 policies were significant only in a few.

Table 5 shows that SD beliefs, TID beliefs, and harm perceptions of smoking were all associated with the total number of tobacco control policies supported in current non-smokers. In current smokers, however, the corresponding associations for TID beliefs and harm perceptions were weaker (Ps for interaction < 0.001) than those in current non-smokers, and SD beliefs were not associated with the total number of policies supported (P for interaction < 0.001). 

## 4. Discussion

We found that support for tobacco control policies in Hong Kong adolescents was weak to moderate. Of the 14 policy options in our questionnaire, 10 were supported by less than 40%, and only one was supported by over 50%. “Stepping up efforts in combating illicit cigarettes” was also supported by almost half of the adolescents, which may have been due to the tobacco industry’s perennial use of illicit cigarettes as an excuse to resist various measures, such as tax increase and larger pictorial warnings, and the resulting media attention on the issue. The public may therefore be particularly familiar with the problem of illicit cigarettes and be supportive of stronger efforts to combat them. Stronger support for tobacco control was found in New Zealand adolescents in a 2012 survey that included four policy options: reducing retail outlets, banning the sale in ten years, extending smokefree areas, and increasing prices [19]. For example, while “banning smoking around children in public places” was supported by 38.8% in our study, “banning smoking in all outdoor places where young people go” was supported by 59% in New Zealand. The differences may have been related to the tobacco control climate in the two settings. In Hong Kong, high-profile policy changes had not been made for several years before the survey, and no large-scale anti-tobacco campaigns were held. In New Zealand, however, the survey was conducted shortly after the introduction of pictorial health warnings, and a youth-oriented mass media anti-tobacco campaign had also been running continuously for several years [19]. The inadequate amount of policy support in Hong Kong adolescents warrants attention. Questions assessing support for tobacco control policies should be included in future tobacco surveys in adolescents. 

We found that, for most of the tobacco control policies, support in girls was stronger than that in boys. On average, girls also supported more policies than boys. Such a difference between the sexes was consistent with the results of previous research in adults [20,21]. Although age was not associated with the number of policies supported, support for most of the policies decreased with age, which may reflect the urge for free choice in older adolescents. Across smoking status, we found a consistent pattern in support for the 14 tobacco control policies—“never”, “ever, non-current”, and “current” smokers displayed the strongest, intermediate, and weakest amounts of support, respectively. Research in adults showed similar patterns [13]. In our study, the levels of support in “ever, non-current” smokers were generally closer to those in “never” smokers, than those in “current” smokers. This might have been due to the high proportion of experimental smokers in “ever, non-current” smokers in our sample. Having fewer co-residing smokers was associated with supporting more policies. Having fewer smoking peers was also associated with supporting more policies, but only in current non-smokers. These findings suggest that family smoking may lower adolescent support for tobacco control policies, and that peer smoking may have a similar effect in current non-smokers. 

We found that SD beliefs, TID beliefs, and harm perceptions of smoking were associated both with support for individual tobacco control policies and with the total number of policies supported in current non-smokers. In current smokers, the corresponding associations were less consistent or weaker. 

Our study is one of the few that explored the role of SD or TID in people’s support for tobacco control policies. The previous two studies also, at least in part, supported the associations of SD or TID with policy support [10,11]. These findings suggest that pushing tobacco use outside the realm of normal practice and exposing the tobacco industry’s malpractices can increase public support for regulations of tobacco products and the industry. Interestingly, we found that, among the associations of TID beliefs with support for individual tobacco control policies, the strongest was for “banning smoking scenes in movies which people under 18 can watch” in both current non-smokers and current smokers. An explanation for this is that adolescents’ perception that the tobacco industry had a role in smoking scenes in movies (presumably reflected in the measurements of TID beliefs) had a particularly strong effect on their support for banning smoking scenes in movies.

The associations between harm perceptions of smoking and support for tobacco control policies in our study were also consistent with previous research in adults [10,11,12,13,14], suggesting that communication of the harms of smoking may also increase policy support. Intervention programmes and trials that involve SD or communication of harmful effects may provide research opportunities to further investigate the role of these interventions in policy support for tobacco control. In addition, it should be noted that the effect estimates in our study were non-standardized. Thus, the estimates for different study factors should not be compared to infer their relative contribution to the outcomes. 

The interaction effects between current smoking and the study factors (SD beliefs, TID beliefs, and harm perceptions of smoking) were non-significant in most cases when the outcomes were supportive of individual tobacco control policies. However, such interaction effects were significant when the outcome was the number of policies supported. The continuous outcome, compared with the binary ones, provided more statistical power for the tests of interaction. These findings suggest that the potential effect of the study factors on policy support for tobacco control may be stronger in current non-smokers than in current smokers. Nonetheless, it is important to note that the number of current smokers (*n* = 718) was much smaller than that of current non-smokers (*n* = 13,246) due to the low adolescent smoking prevalence in Hong Kong. In current smokers, of the 42 PRs of support for individual policies, 35 PRs favored policy support (PR >1), although only seven were statistically significant. Many of the non-significant PRs were comparable in magnitudes with those in current non-smokers. Had the sample of current smokers been larger, more PRs favoring policy support might have reached statistical significance. Overall, the findings in current smokers are still encouraging and suggest that, even in populations with higher adolescent smoking prevalence, SD, TID, and communication of the harms of smoking may also help garner support for tobacco control. 

In practice, TID is underused, especially outside the U.S. [9]; yet other support for TID, direct or subtle, still exists. Firstly, it is recommended in the guidelines of the Framework Convention on Tobacco Control [22]. Secondly, TID campaigns in the US were strongly opposed by the tobacco industry [23], and the industry’s internal documents also revealed its intention “to limit the spread of: demonization’ (of the industry) from the developed world to the emerging markets” [24]. Such reactions suggest that TID has passed the “scream test”—an indication of effective measures that constitute a threat to the industry’s interests and legitimacy [9]. Thirdly, without large-scale TID efforts, the public’s awareness of industry tactics may be low. In our sample, more than two-fifths did not believe that the industry tried to get youth to smoke, and only less than one-fifth firmly believed this was the case (not shown in tables). A similar awareness gap was found in a survey of UK adults [25]. These findings suggest that substantial proportions of adolescents and adults still consider the tobacco industry to be quite credible and respectable. Wider and more effective use of TID is warranted, which may also help generate stronger evidence on TID’s effects on public support for tobacco control policies.

While the harmful effects of tobacco have been communicated to the public in many ways, efforts at communicating SD and TID have been more limited. Traditional anti-tobacco education in children and adolescents should be broadened to include SD and TID components, and should correct the overestimation of smoking prevalence, associate smoking with being abnormal, and expose the industry’s malpractices. The industry’s tactics to get young people to smoke should especially be emphasized. In addition, cigarette packets could be used for showing messages and pictures that deformalize tobacco products or the industry. The print media can also be a channel for denormalization. For example, newspaper articles can be used to counter the industry’s misleading arguments against tobacco control policies. Studies of print media tobacco coverage found either no or a small number of articles with themes on SD or TID [4,26]. 

Because of the cross-sectional design, the associations we found might have been affected by residual confounding and reverse causality. Nonetheless, an effect of our study factors (SD beliefs, TID beliefs, and harm perceptions of smoking) on support for tobacco control policies seems plausible, and our study is one of the first that focus on this important issue. Future research with stronger study designs is warranted. Another limitation is the low school-level response rate (36%). However, the recruited schools were not significantly different from those not recruited in districts, mediums of instruction, sources of financial support, and single- or mixed-sex education (Ps for chi-square <0.05). 

## 5. Conclusions

In this paper, we found that the support level for tobacco control policies in Hong Kong adolescents was weak to moderate. SD beliefs, TID beliefs, and harmful perceptions of smoking were associated with support for tobacco control policies in current non-smokers. In current smokers, the corresponding associations were less consistent or weaker.

## Figures and Tables

**Table 1:** Sample characteristics.

Sample Characteristics	N (%) ^a^
**Sex**	
Girls	6799 (48.7)
Boys	7164 (51.3)
**Age in years; Mean (SD) ^b^**	15.0 (1.8)
**Perceived family affluence**	
Relatively poor	915 (6.6)
Poor to average	3556 (25.5)
Average	7958 (57.0)
Average to rich	1329 (9.5)
Relatively rich	205 (1.5)
**Peer smoking**	
None	9676 (69.3)
Less than half	3457 (24.8)
Half or more	830 (5.9)
**Number of co-residing smokers**	
0	8743 (62.6)
1	3890 (27.9)
≥2	1330 (9.5)
**Smoking**	
Never	11,972 (85.7)
Ever, non-current	1243 (8.9)
Current	748 (5.4)
**Social denormalization beliefs ^c^; Mean (SD)**	3.5 (1.1)
**Tobacco industry denormalization beliefs ^c^; Mean (SD)**	3.8 (1.3)
**Harm perceptions of smoking ^d^; Mean (SD)**	2.9 (0.5)
**Total number of tobacco control policies supported ^e^; Mean (SD)**	4.8 (4.6)

^a^ Numbers and percentages unless otherwise stated. ^b^ SD = standard deviation. ^c^ Range 0–6, with greater values indicating stronger denormalization beliefs. ^d^ Range 0–3, with greater values indicating stronger harm perceptions. ^e^ Range 0–14.

**Table 2:** Support (%) for tobacco control policies by sex, age, and smoking status.

Tobacco Control Policies	Overall	Sex			Age (in years)				Smoking Status			
		Girls	Boys	*p*-Value ^a^	≤13	14–15	≥16	*p*-Value ^a^	Never	Ever, Non-Current	Current	*p*-Value ^a^
Ban smoking within 5 m from the entrance of a building	39.5	41.0	38.0	<0.001	41.2	38.5	39.1	NS	42.0	34.1	8.4	<0.001
Ban smoking at transport waiting areas	54.1	61.5	46.9	<0.001	51.9	52.8	56.3	<0.001	56.5	51.7	19.7	<0.001
Ban smoking around children in public places	38.8	43.7	34.2	<0.001	40.4	37.8	38.6	NS	40.7	38.1	10.2	<0.001
Ban smoking at home when a child is present	34.9	38.4	31.5	<0.001	38.3	35.1	32.7	<0.001	36.7	32.2	11.1	<0.001
Ban smoking in a private car when a child is present	35.9	39.7	32.3	<0.001	37.8	35.5	35.0	0.04	37.6	34.1	11.7	<0.001
Show health warning texts and pictures on at least 75% of cigarette packet surfaces, and ban the display of trademarks	34.0	35.4	32.6	0.001	38.1	32.6	32.4	<0.001	35.4	31.5	15.5	<0.001
Stronger health warning pictures on cigarette packets	34.9	37.0	32.9	<0.001	38.5	33.9	33.5	<0.001	36.7	31.3	11.3	<0.001
Change the health warning pictures on cigarette packets every 1–2 years	23.6	23.4	23.8	NS	25.9	23.0	22.7	0.001	24.6	23.1	9.0	<0.001
Show smoking cessation hotlines on cigarette packets	35.0	36.1	33.9	0.005	39.5	34.7	32.5	<0.001	36.6	31.0	15.9	<0.001
Ban the display of tobacco products at point of sale	36.7	40.7	33.0	<0.001	40.0	36.5	34.9	<0.001	39.4	29.3	7.0	<0.001
Ban smoking scenes in movies which people under 18 can watch	17.6	18.2	17.1	NS	21.6	15.9	16.6	<0.001	19.0	12.3	4.1	<0.001
Ban smoking in people born since 2010	23.0	22.0	23.9	0.04	25.5	22.8	21.6	<0.001	24.0	18.3	14.9	<0.001
Further increase tobacco tax	45.7	49.3	42.2	<0.001	45.0	44.3	47.1	NS	48.4	38.2	14.8	<0.001
Step up efforts in combating illicit cigarettes	49.7	54.6	45.0	<0.001	49.0	49.0	50.6	NS	52.6	42.4	15.0	<0.001

^a^*p*-values produced by chi-square tests. NS = Non-significant.

**Table 3:** β coefficients (95% confidence intervals) of the total number of tobacco control policies supported in relation to sociodemographic and smoking-related characteristics.

Sociodemographic and Smoking-Related Characteristics	Number of Tobacco Control Policies Supported				P for Interaction	
	Current Non-Smokers		Current Smokers			
	Crude β (95% CI)	Adjusted β (95% CI)	Crude β (95% CI)	Adjusted β (95% CI)	Crude β	Adjusted β
**Sex**						
Boys	Ref	-	Ref	-		
Girls	0.62 *** (0.44, 0.80)	-	0.50 *** (0.22, 0.78)	-	NS	-
**Age**						
≥16	Ref	-	Ref	-		
14–15	−0.10 (−0.33, 0.14)	-	−0.03 (−0.39, 0.32)	-	NS	-
≤13	0.23 (−0.14, 0.61)	-	0.05 (−0.21, 0.30)	-	NS	-
**Perceived family affluence**						
Relatively rich	Ref	-	Ref	-		
Average to rich	0.78 (−0.22, 1.77)	-	0.66 (−0.59, 1.91)	-	NS	-
Average	0.47 (−0.49, 1.44)	-	−0.13 (−1.05, 0.79)	-	NS	-
Poor to average	0.77 (−0.15, 1.69)	-	0.03 (−0.86, 0.93)	-	NS	-
Relatively poor	1.06 * (0.04, 2.08)	-	−0.17 (−1.15, 0.80)	-	NS	-
**Peer smoking**						
Half or more	Ref	Ref	Ref	Ref		
Less than half	1.55 *** (1.16, 1.95)	1.08 *** (0.74, 1.42) ^a^	0.28 (−0.15, 0.71)	0.28 (−0.16, 0.72) ^b^	<0.001	<0.001 ^b^
None	2.27 *** (1.79, 2.76)	1.38 *** (0.96, 1.81) ^a^	0.31 (−0.24, 0.86)	0.31 (−0.26, 0.87) ^b^	<0.001	<0.001 ^b^
**Number of co-residing smokers**						
≥2	Ref	Ref	Ref	Ref		
1	0.33 * (0.05, 0.61)	0.33 * (0.04, 0.61) ^c^	0.43 * (0.01, 0.84)	0.44 * (0.04, 0.85) ^c^	NS	NS ^c^
0	0.73 *** (0.43, 1.04)	0.74 *** (0.43, 1.04) ^c^	0.47 (−0.02, 0.96)	0.46 (−0.02, 0.93) ^c^	NS	NS ^c^

* *p* < 0.05; *** *p* < 0.001. NS = Non-significant. ^a^ With adjustment of all the other sociodemographic and smoking-related characteristics in the table, smoking (“never” vs. “ever, non-current”), SD beliefs, TID beliefs, and harm perceptions of smoking. ^b^ With the same adjustment in a, but without the adjustment of smoking (“never” vs. “ever, non-current”). ^c^ With adjustment of perceived family affluence.

**Table 4:** Adjusted prevalence ratios (95% confidence intervals) ^a^ of support for tobacco control policies in relation to SD beliefs ^b^, TID beliefs ^c^, and harm perceptions of smoking ^d^.

Support for Tobacco Control Policies	Current Non-Smokers			Current Smokers			P for Interaction ^e^		
	SD Beliefs ^b^	TID Beliefs ^c^	Harm Perceptions of Smoking ^d^	SD Beliefs ^b^	TID Beliefs ^c^	Harm Perceptions of Smoking ^d^	SD Beliefs ^b^	TID Beliefs ^c^	Harm Perceptions of Smoking ^d^
Ban smoking within 5 m from the entrance of a building	1.10 *** (1.08, 1.13)	1.12 *** (1.11, 1.14)	1.18 *** (1.09, 1.27)	1.27 ** (1.08, 1.49)	1.11 (0.91, 1.36)	1.10 (0.90, 1.34)	NS	NS	NS
Ban smoking at transport waiting areas	1.07 *** (1.05, 1.09)	1.05 *** (1.04, 1.07)	1.23 *** (1.15, 1.31)	1.08 (0.96, 1.21)	1.05 (0.96, 1.15)	1.20 (0.99, 1.46)	NS	NS	NS
Ban smoking around children in public places	1.07 *** (1.04, 1.11)	1.12 *** (1.10, 1.13)	1.35 *** (1.22, 1.48)	1.09 (0.85, 1.40)	1.14 (0.97, 1.34)	1.31 (0.94, 1.82)	NS	NS	NS
Ban smoking at home when a child is present	1.08 *** (1.06, 1.11)	1.13 *** (1.11, 1.16)	1.22 *** (1.12, 1.32)	0.81 * (0.66, 1.00)	1.09 (0.99, 1.22)	1.43 ** (1.10, 1.86)	0.008	NS	NS
Ban smoking in a private car when a child is present	1.06 *** (1.04, 1.09)	1.11 *** (1.09, 1.13)	1.33 *** (1.21, 1.46)	1.11 (0.97, 1.26)	1.01 (0.90, 1.13)	1.25 (0.90, 1.72)	NS	NS	NS
Show health warning texts and pictures on at least 75% of cigarette packet surface, and ban the display of trademarks	1.08 *** (1.06, 1.10)	1.12 *** (1.10, 1.14)	1.21 *** (1.11, 1.31)	0.89 (0.78, 1.01)	1.09 (0.96, 1.25)	1.26 * (1.03, 1.53)	0.002	NS	NS
Stronger health warning pictures on cigarette packets	1.09 *** (1.07, 1.11)	1.11 *** (1.09, 1.12)	1.24 *** (1.13, 1.36)	1.03 0.87, 1.22)	1.04 (0.94, 1.14)	1.20 (0.94, 1.55)	NS	NS	NS
Change the health warning pictures on cigarette packets every 1–2 years	1.12 *** (1.09, 1.15)	1.14 *** (1.12, 1.17)	1.19 *** (1.07, 1.32)	0.94 (0.78, 1.13)	1.11 (0.98, 1.25)	1.34 (0.99, 1.81)	0.01	NS	NS
Show smoking cessation hotlines on cigarette packets	1.09 *** (1.07, 1.12)	1.10 *** (1.08, 1.12)	1.26 *** (1.16, 1.37)	1.12 (0.94, 1.34)	1.06 (0.94, 1.20)	0.96 (0.74, 1.24)	NS	NS	NS
Ban the display of tobacco products at point of sale	1.10 *** (1.08, 1.13)	1.18 *** (1.15, 1.20)	1.27 *** (1.16, 1.39)	1.16 (0.92, 1.48)	1.15 (0.97, 1.36)	1.34 (0.88, 2.05)	NS	NS	NS
Ban smoking scenes in movies which people under 18 can watch	1.12 *** (1.08, 1.16)	1.27 *** (1.23, 1.31)	1.19 ** (1.06, 1.34)	0.97 (0.78, 1.21)	1.22 * (1.02, 1.47)	0.84 (0.56, 1.26)	NS	NS	NS
Ban smoking in people born since 2010	1.14 *** (1.09, 1.18)	1.25 *** (1.22, 1.27)	1.21 *** (1.11, 1.32)	1.09 (0.96, 1.23)	1.10 * (1.00, 1.21)	1.24 (0.98, 1.57)	NS	0.006	NS
Further increase tobacco tax	1.09 *** (1.07, 1.11)	1.11 *** (1.10, 1.13)	1.25 *** (1.16, 1.34)	1.22 *** (1.08, 1.38)	1.05 (0.93, 1.20)	1.03 (0.84, 1.26)	NS	NS	NS
Step up efforts in combating illicit cigarettes	1.07 *** (1.04, 1.09)	1.07 *** (1.05, 1.08)	1.29 *** (1.19, 1.41)	0.95 (0.84, 1.08)	1.02 (0.94, 1.12)	1.42 *** (1.16, 1.73)	0.04	NS	NS

* *p* < 0.05; ** *p* < 0.01; *** *p* < 0.001. NS = Non-significant. ^a^ With mutual adjustment of SD beliefs, TID beliefs, and harm perceptions of smoking and with adjustment of age, sex, perceived family affluence, peer smoking, and the number of co-residing smokers. In current non-smokers, smoking (“never” vs. “ever, non-current”) was also adjusted. Adjusted prevalence ratios were for per unit increase in the study factors. ^b^ Range 0–6, with greater values indicating stronger social denormalization beliefs. ^c^ Range 0–6, with greater values indicating stronger tobacco industry denormalization beliefs. ^d^ Range 0–3, with greater values indicating stronger harm perceptions. ^e^ With the same adjustment as in current smokers.

**Table 5:** Adjusted β coefficients (95% confidence intervals) ^a^ of the total number of tobacco control policies supported in relation to SD beliefs ^b^, TID beliefs ^c^, and harm perceptions of smoking ^d^.

Tobacco Denormalization Beliefs or Harm Perceptions	Number of Tobacco Control Policies Supported		P for Interaction ^e^
	Current Non-Smokers	Current Smokers	
**SD beliefs ^b^**	0.44 *** (0.35, 0.52)	0.05 (−0.09, 0.19)	<0.001
**TID beliefs ^c^**	0.57 *** (0.51, 0.63)	0.12 * (0.01, 0.24)	<0.001
**Harm perceptions of smoking ^d^**	0.81 *** (0.62, 1.00)	0.25 *** (0.13, 0.37)	<0.001

* *p* < 0.05; *** *p* < 0.001. NS = Non-significant. ^a^ With mutual adjustment of SD beliefs, TID beliefs, and harm perceptions of smoking, and with adjustment of age, sex, perceived family affluence, peer smoking, and the number of co-residing smokers. In current non-smokers, smoking (“never” vs. “ever, non-current”) was also adjusted. ^b^ Range 0–6, with greater values indicating stronger social denormalization beliefs. ^c^ Range 0–6, with greater values indicating stronger tobacco industry denormalization beliefs. ^d^ Range 0–3, with greater values indicating stronger harm perceptions. ^e^ With the same adjustment as in a in current smokers.
